# Light triggered detection of aminophenyl phosphate with a quantum dot based enzyme electrode

**DOI:** 10.1186/1477-3155-9-46

**Published:** 2011-10-07

**Authors:** Waqas Khalid, Gero Göbel, Dominik Hühn, Jose-Maria Montenegro, Pilar Rivera-Gil, Fred Lisdat, Wolfgang J Parak

**Affiliations:** 1 Fachbereich Physik and WZMW, Philipps Universität Marburg, Germany; 2 Biosystems Technology, University of Applied Sciences Wildau, Wildau, Germany

## Abstract

An electrochemical sensor for *p*-aminophenyl phosphate (*p*APP) is reported. It is based on the electrochemical conversion of 4-aminophenol (4AP) at a quantum dot (QD) modified electrode under illumination. Without illumination no electron transfer and thus no oxidation of 4AP can occur. *p*APP as substrate is converted by the enzyme alkaline phosphatase (ALP) to generate 4AP as a product. The QDs are coupled via 1,4-benzenedithiol (BDT) linkage to the surface of a gold electrode and thus allow potential-controlled photocurrent generation. The photocurrent is modified by the enzyme reaction providing access to the substrate detection. In order to develop a photobioelectrochemical sensor the enzyme is immobilized on top of the photo-switchable layer of the QDs. Immobilization of ALP is required for the potential possibility of spatially resolved measurements. Geometries with immobilized ALP are compared versus having the ALP in solution. Data indicate that functional immobilization with layer-by-layer assembly is possible. Enzymatic activity of ALP and thus the photocurrent can be described by Michaelis- Menten kinetics. *p*APP is detected as proof of principle investigation within the range of 25 μM - 1 mM.

## Introduction

Colloidal quantum dots (QDs), which are fluorescent semiconductor nanoparticles, have recently brought impact to various disciplines, as has been highlighted in various review articles [1-5]. QDs have been recently discussed also as new building blocks for the construction of electrochemical sensors [6-12]. Upon optical illumination (below the wavelength of the first exciton peak QDs have a a continuous absorption spectrum, with a local maximum at the exciton peak [13]) electron hole pairs are generated inside QDs. Due to these charge carriers electrons can be transferred to or from the QDs. QDs thus can be oxidized/reduced and can serve as light-controlled redox active element and can be integrated in electrochemical signal chains [9,14-16]. The key advantage hereby is that the redox reaction of the QD surface can be virtually switched on and off by light. QD have been also used as elements of signal transduction of enzymatic reactions [17,18].

In the present work we wanted to apply QDs as light-controlled redox active element for the enzymatic detection of *p*-aminophenyl phosphate (*p*APP) with alkaline phosphatase (ALP). ALP is a widely used enzyme in bioanalysis as it has a high turnover rate and broad substrate specificity [19]. The enzyme is particularly interesting as label for immunoassays [20,21]. Very sensitive substrate recycling schemes have been also reported [22,23]. Four different groups of substrates are known for ALP: i) ß-glycerophosphate and hexose phosphate [24-26], ii) phenyl phosphate [27,28] and ß-naphthyl phosphate [29], iii) *p*-nitrophenyl phosphate [30] and phenolphthalein diphosphate [31,32], 4-methyl-umbellipheryl phosphate [33] and *p*-aminophenyl phosphate (*p*APP) [34], and iv) phosphoenol pyruvate [35]. Electrochemical detection has been reported for a number of ALP substrates [36,37], in particular for phenyl phosphate. However, *p*APP is claimed to be a better substrate for ALP than phenyl phosphate, as its product 4-aminophenol (4AP) is more easily oxidizable than phenol, which is the product of phenyl phosphate, as it does not foul the electrode even at higher concentrations, and as it has a rather reversible electrochemical behavior [34]. For this reason we chose *p*APP as substrate in the present study. Readout of the enzymatic reaction was performed with the QD-modified electrode [6]. We hereby put particular interest in the way of immobilization of ALP on the electrode. In previous work the enzymes were suspended in the solution above the sensor electrode [6,9]. Here we go a step further and directly immobilize the enzyme on the QD-modified electrode. This was done in order to investigate whether a specific enzymatic reaction can be coupled with a photoinitiated reaction at a QD modified electrode in a way that the recognition element is integrated with the transducer. The potential advantage of light-triggered detection would be the possibility of spatially resolved detection [38-41]. Only at the illuminated parts of the electrode a photocurrent signal is induced. By having different enzymes immobilized at different regions of the electrode they could be selectively addressed by illumination. Thus, two key elements of this study are the following. First, instead of using enzymes in solution as in previous studies we demonstrate that enzymatic reactions can also be followed when enzymes are immobilized on the sensor surface, which is a requirement for potential spatially resolved analysis. Second, we investigate how the way of immobilization influences the sensing properties.

## Materials and Methods

Materials: CdS QDs were grown via thermal decomposition of precursors under the presence of organic surfactant molecules following published procedures [42]. 1,4-benzenedithiol (BDT) was purchased from TCI Europe, Belgium. Chloroform, toluene, methanol, acetone, ethanol, sodium sulfide (nanohydrate), alkaline phosphatase (from bovine intestinal mucosa type VII S), 4-nitrophenyl phosphate disodium hexahydrate, 4-aminophenol (4AP), phosphate buffer, sodium poly(styrene sulfonate) (PSS, M_w _= 56,000), poly(allylamine hydrochloride) (PAH, M_w _= 70,000), and potassium ferri/ferro cyanide were purchased from Sigma Aldrich and used without further purification. All aqueous solutions were prepared using 18 MΩ ultra purified water. The electrochemical measurement cells and electronics have been described in a previous publication [43] and comprised a home built potentiostat, an Ag/AgCl reference electrode (#MF 2078 RE-6 from BASi, UK), and a lock-in amplifier (EG&G Princeton Applied Research model # 5210). Illumination was done with a xenon lamp (PTI model A-1010 arc lamp housing, UXL-75XE Xenon Lamp from USHIO, powered by PTI LPS-220) modulated by an optical chopper (Scitec instruments).

**Figure 1:**
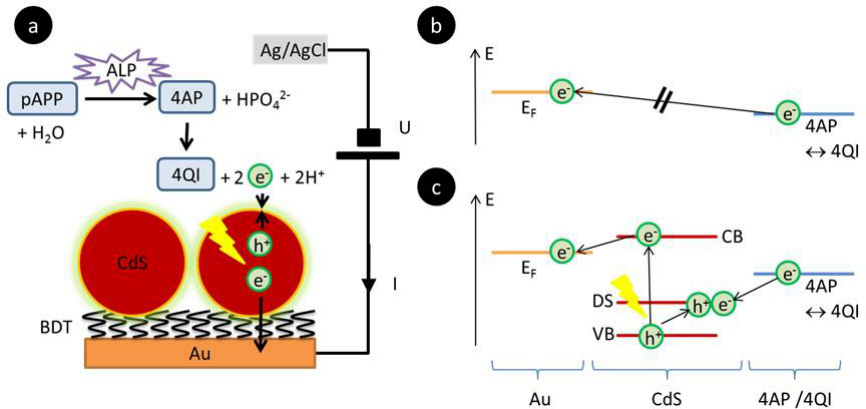
**Detection principle and redox schemes**. a) Sketch of the detection scheme. A bias voltage U is applied to a Au electrode versus an Ag/AgCl reference electrode in the bath solution. The Au electrode is coated with CdS QDs which are attached via a BDT layer. *p*APP is in solution degraded by ALP to 4AP. Upon illumination of the QDs electron hole pairs are generated. This leads to oxidation of 4AP to 4QI on the QD surface, whereby electrons are transferred to the QD. Electrons are passed to the Au electrode and can be detected as oxidation current I. b) Without QDs as redox mediator oxidation of 4AP can't happen in case the bias potential U is not positive enough. Energy levels E are shown. For oxidation the Fermi level E_F _of the Au electrode would need to be lower than the energy level at which electrons upon oxidation of 4AP are released. c) Illuminated QDs can act as redox mediator. Defect states (DS) at the QD surface (which are energetically above the valance band VB) prevent light generated electron hole pairs from immediate recombination. In this way electrons resulting from the oxidation of 4AP to 4QI can be transferred to the DS of the QD. In turn electrons from the conduction band (CB) can be drained via the BDT layer to the gold electrode, which is detected as oxidation/photocurrent.

Immobilization of QDs: CdS QDs were immobilized on top of gold electrodes following a previously published protocol [43], cfg. Figure 1. First, the gold electrodes (Au film evaporated on glass chips) were cleaned by sonication toluene for five minutes. For cleaning the cyclic voltammetry (CV) of the gold electrode was performed in 1 M NaOH for 20 minutes within the potential limits of -0.8 V < U < +0.2 V, and later in 0.5 M H_2_SO_4 _for 30 minutes within the potential limits of -0.2 V < U < 1.6 V (the CV curves are shown in Additional File 1). After cleaning, the gold electrodes were placed in a solution of 50 mM BDT dissolved in toluene for 24 hours. This resulted in a self assembled monolayer of BDT on the gold surface due to formation of thiol-gold bonds. In the next step CdS QDs dissolved in toluene (typically with a first exciton peak around 380 nm, concentration around 140 μM) were spin coated at a speed of 6000 rpm on top of the BDT coated gold electrodes. After spin coating the gold electrodes were rinsed twice with toluene to remove the excess of QDs.

Confirmation of QDs immobilization: Immobilization of CdS QDs on top of the Au electrodes was performed with current measurements. CVs were recorded before and after immobilization of BDT and QDs on top of gold electrodes with Fe^3+^/Fe^2+ ^as redox couple in solution [43]. While on bare gold electrodes the typical oxidation and reduction currents could be observed these were not visible in the case of gold electrodes coated with BDT and QDs (see Additional File 1 for data). Alternatively current at fixed bias voltage was recorded for gold electrodes before and after immobilization of BDT and QDs, while illumination was switched on and off. In the case of QDs present on top of the Au electrode a photocurrent could be measured under illumination (date are shown in Additional File 1)

**Figure 2:**
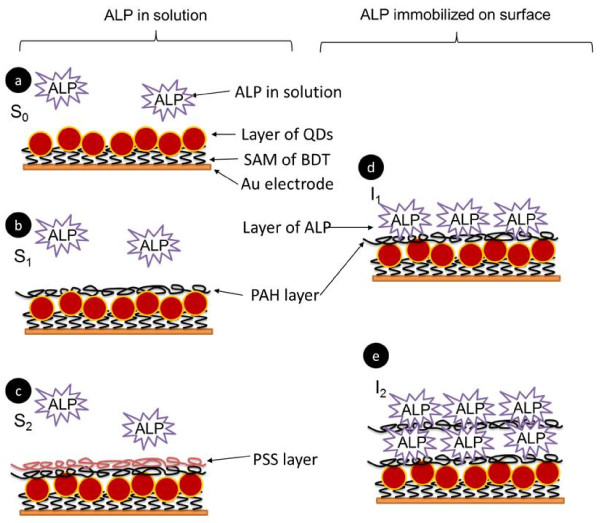
**Different geometries for introducing ALP**. ALP can be either suspended in solution (S) or immobilized at the electrode surface (I). CdS QDs have been attached to the electrode surface via a BDT layer and spin coating. On top of the QD layer optionally polyelectrolyte layers out of PAH and PSS are added. Hereby i is the number of polyelectrolyte layers: S_0_, S_1_, S_2_, I_0_, I_1_. a) S_0_: immobilization of QDs via spin coating with ALP in solution. b) S_1_: a single layer of PAH is added on top of S_0_. c) S_2_: a layer of PSS is immobilized on top of S_1_. d) I_1_: ALP is immobilized on to of S_1_. e) I_2_: A second double layer of PAH and ALP is immobilized on top of I_1_.

Solubilized versus immobilized enzymes: In order to observe the enzymatic reaction of ALP and *p*APP the enzyme ALP was either directly added to the bath solution (S) or immobilized on top of the QDs layer (I). All geometries are depicted in Figure 2. In the simplest case (S_0_) the Au electrodes with spin coated QDs layer were directly used without further modification. For the next geometry (S_1_) a polyelectrolyte layer of PAH was coated on top of the CdS QDs layer mediated by electrostatic attraction by immersing the QDs coated Au electrode in a solution of PAH for 5 minutes (0.02 M monomer concentration, pH = 6.5, 0.5 M NaCl) [43,44]. Unbound excess PAH was removed by rinsing. PAH is positively charged. We speculate that the QDs layer is not tight so that PAH is attracted by the negatively charged underlying BDT monolayer. Stability after rinsing confirmed stable deposition of PAH. To this configuration a second polyelectrolyte layer (S_2_) of PSS could be added by immersing the PAH coated QDs-Au electrode (S_1_) for 5 minutes in a solution of PSS (0.02 M monomer concentration, pH = 6.5, 0.5 M NaCl), followed by a rinsing step to remove unbound PSS. PSS is negatively charged and thus electrostatically attracted by the PAH layer [44]. In all three geometries (S_0_, S_1_, S_2_) ALP was added directly to the solution on top of the electrode without any direct attachment. We also tried to directly immobilize ALP on the electrodes. For this purpose QDs coated Au electrodes were first modified with a PAH layer, leading to a positively charged surface (S_1_). To this negatively charged ALP [45,46] was added by 5 minutes immersion in a solution of ALP (120 units/ml, pH = 7.8, 10 mM phosphate buffer). Attachment of ALP to PAH was mediated by electrostatic interaction (I_1_). In order to increase the amount of immobilized ALP, the coating procedure was repeated (I_2_). The electrodes with one layer of ALP were immersed again for 5 minutes in a solution of PAH, followed by rinsing, and then for 5 minutes in a solution of ALP followed by rinsing. This step-wise multilayer assembly mediated by electrostatic interaction [44] lead to two layers of ALP on top of the QD coated Au electrodes. Layer-by-layer assembly was confirmed with fluorescence labeled polyelectrolytes (data see Additional File 1).

**Figure 3:**
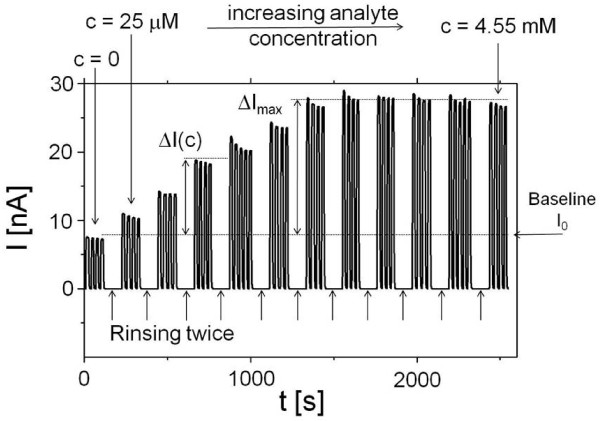
**Detection principle of dose response curves**. A constant bias U = +200 mV is applied and current I is detected. Hereby illumination is switched on and off with a shutter. During the periods without illumination no current can flow. The base line current I_0 _is detected. After 2 rinsing steps analyte is added (in this case 4AP dissolved in 25% methanol and 75% phosphate buffer at pH 5, geometry S_o_) and the respective photocurrent I is recorded in phosphate buffer with final pH = 7.8. This process is repeated while successively adding more analyte (in the present example 4AP concentration was increased from 25 μM to 4.55 mM). The respective oxidation current response ΔI(c) for each analyte concentration c is derived by subtracting the base line I_0 _from the detected photocurrent I(c). The dose response curve for the present example is displayed in Figure 4a.

**Figure 4:**
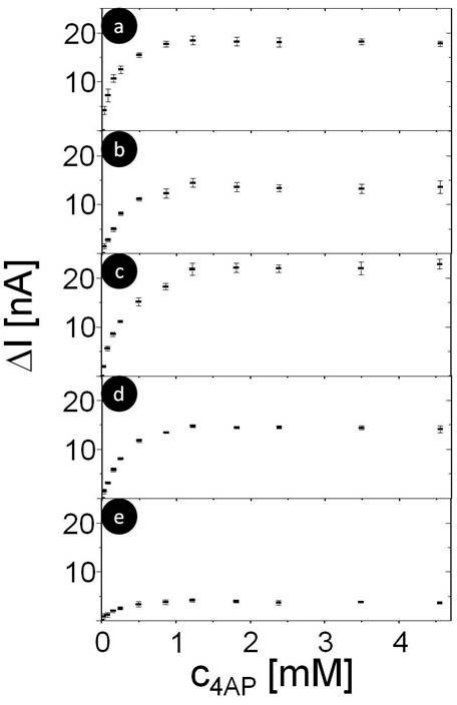
**Dose response curve for detection of 4AP (originally dissolved in 25% methanol and 75% phosphate buffer pH 5) as recorded in phosphate buffer pH 7.8 at bias potential of +200 mV for geometries a) S**_**0**_**, b) S**_**1**_**, c) S**_**2**_**, d) I**_**1**_**, e) I**_**2**_. The resulting photocurrent I is plotted versus the concentration c of 4AP.

**Figure 5:**
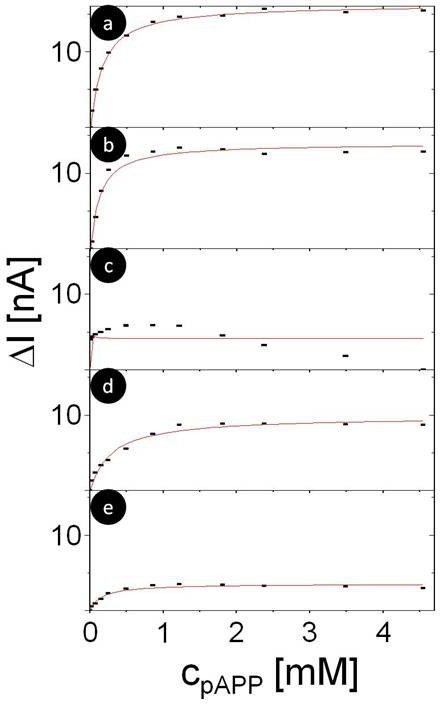
**Dose response curve for detection of *p*APP as recorded in phosphate buffer pH 7.8 at bias potential of +200 mV under the presence of ALP (120 units per 2 ml in case of geometry S) for geometries a) S**_**0**_**, b) S**_**1**_**, c) S**_**2**_**, d) I**_**1**_**, e) I**_**2**_. The resulting photocurrent I is plotted versus the concentration c of *p*APP. The solid line in each of the curves indicates a fit with the Michaelis-Menten equation. Values are displayed in Table 1

Electrochemical measurements of dose-response curves: A constant bias voltage U was applied and the base line photocurrent I_0 _was measured in phosphate buffer solution (pH 7.8) by switching illumination on and off with mechanical shutter, see Figure 3. Then the electrochemical cell was rinsed twice and a known amount of 4AP (product of ALP) or *p*APP (substrate for ALP) was added and the photocurrent I was measured again. Also hereby illumination was switched on and off several times with a mechanical shutter. For the next measurement the cell was again rinsed twice, an increasing amount of 4AP or *p*APP was added, and the photocurrent I was measured while switching on and off the illumination. With this procedure the response in photocurrent ΔI(c) = I(c) - I_0 _to different concentrations of 4AP or *p*APP was determined, see Figure 3. The resulting dose-response curves are plotted in Figures 4-5. It has to be noted that after each excitation there is a slight decrease in photocurrent, which we have previously ascribed to degradation of the QDs layer [43]. Polyelectrolyte layers above the QDs layer have been demonstrated to increase stability [43].

## Results and Discussion

**Figure 6:**
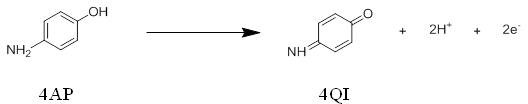
**Oxidation reaction of 4-aminophenol (4AP) to 4-quinoneimine (4QI)**.

Detection of 4AP and sensor principle: First we have investigated whether the CdS modified gold electrode can be used as transducer to the analysis of 4AP - the reaction product of ALP reaction. For this purpose the electrode potential U was varied and the current I was measured under pulsed illumination. A clear response of the photocurrent to the presence of 4AP was found indicating that the QDs electrode provides a suitable surface for 4AP oxidation (cfg. Figure 3). Since the electrochemical behavior of 4AP is well known, the reaction is shown in Figure 6.

**Figure 7:**
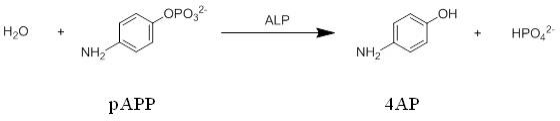
**Hydrolysis reaction of *p*-aminophenol phosphate to 4-aminophenol catalyzed with alkaline phosphatase**.

A maximum of photocurrent was detected for an applied bias potential of +200 mV against Ag/AgCl, 3M KCL (data are shown in Additional File 1). For this reason all following measurements were performed at fixed bias U = +200 mV. On the basis of the sensitivity of the QD electrode for 4AP, we wanted to construct a photoelectrochemical sensor. A sketch of our sensor concept is depicted in Figure 1. In presence of ALP *p*APP is hydrolyzed to 4AP and HPO_4_^2- ^(cfg. Figure 7) which is subsequently converted at the electrode under illumination.

The actual sensor electrode was composed out of QDs which were coupled via a 1,4-benzenedithiol (BDT) layer on top of a gold film electrode. A bias voltage U = +200 mV was applied and the corresponding current I was recorded. Upon illumination of the QDs, electron-hole pairs were generated. Electron transfer could take place in between CdS QDs and the 4AP/QI - redox couple in solution and in between the QDs and the electrode. Thus, the QDs could be used as a light-triggered interlayer to transfer electrons from the redox couple, present in solution to the electrode. The energetical situation of the electron transfer pathway is depicted in Figure 1b/c. 4AP could be only oxidized to 4QI if the two released electrons could be transferred to an energetically lower level. In case the bias U applied to a gold electrode was not positive enough (i.e. its Fermi level was above the energy of the 4AP/4QI redox couple), no oxidation of 4AP could occur (cfg. Figure 1b). However, if at the same bias illuminated QDs were used oxidation of 4AP was possible (cfg. Figure 1c). Upon illumination, electrons in the QDs were excited from the valence band (VB) to the conduction band (CB), resulting in electrons (e^-^) and holes (h^+^). The holes were trapped in defect states (DS) [47] at the surface of the QDs. 4AP could now be oxidized to 4QI upon transferring the electrons to the QDs where they recombined with the holes. In turn, electrons were transferred from the CB of the QDs to the gold electrode, thus creating an oxidation current I.

In order to realize this signal chain in a sensor format (with the potential possibility of spatially resolved detection) the enzyme needed to be immobilized on the photosensitive electrode. The layer by layer approach in depositing protein molecules is a very favorable technique since it allows control on the deposited amount in one layer but also in the whole assembly by the number of deposition steps [48,49]. In order to deposit ALP, the positively charged polyelectrolyte PAH was used here. We have investigated ALP as a monolayer but also as bilayer. In order to mimic the influence of the charge situation we have studied the effect of the polyelectrolyte alone on the sensing behavior. Figure 2 summarizes the different systems which have been analyzed on the way to a sensing electrode. To ensure high sensitivity for 4AP detection, the influence of protein and polyelectrolyte interlayers on the photocatalytic oxidation of 4AP were investigated. The oxidation current for different 4AP concentrations was determined for all 5 geometries shown in Figure 2. For each geometry a dose response curve was generated, see Figure 4. Data demonstrate that the concentration of 4AP can be reasonably detected within the ranges of 25 μM to around 1.5 mM. For 4AP concentrations larger than 1.5 mM the photocurrent response is saturated for all geometries. However, there was a significant difference in the maximum response of the oxidation current. The maximum photocurrents ΔI_max _at saturation are displayed in Table 1. For geometry S_2 _the higher current probably might be due to electrostatic attraction of negatively charged PSS and 4AP. For geometry I_2 _the photocurrent response is smaller than for the other geometries (Figure 4e). This might be ascribed to a rather dense assembly of ALP with PAH hindering 4AP to reach the QDs modified electrode. At any rate, the data show that the polyelectrolyte used and the immobilized protein still allow the conversion of the reaction product of ALP. Thus another important precondition for the sensor construction seems to be fulfilled.

**Table 1:** Oxidation currents for the different geometries

Geometry	ΔI_max _[nA] direct detection of 4AP	ΔI_max _[nA] direct detection of *p*APP	ΔI_max _[nA] enzymatic reaction	K_M _[mM]
S_0_	18.4	2.1	16.3	0.16

S_1_	14.4	2.1	14.0	0.12

S_2_	21.8	-	-	-

I_1_	14.8	-	9.8	0.29

I_2_	4.1	-	3.5	0.15

Maximum oxidation currents are recorded for different geometries S_0_, S_1_, S_2_, I_1_, I_2 _as recorded in phosphate buffer with pH = 7.8. Data are shown for detection of 4AP (cfg. Figure 4), *p*APP (cfg. Additional File 1), and detection of 4AP after enzymatic degradation of *p*APP with ALP (cfg. Figure 5). In the case of the enzymatic reaction also the Michaelis-Menten constant K_M _is given.

Detection of *p*-aminophenyl phosphate: As an experimental complication it has to be pointed out that *p*APP has limited stability, since *p*APP decomposes slowly in alkaline solution [50]. In order to be sure to test the enzyme activity on the CdS electrode, *p*APP has also been investigated with the 3 different geometries given in Figure 2 (without the enzyme). Only a very small response of about 1-2 nA was obtained (cfg. Table 1 and Additional File 1). This is an order of magnitude lower than the response to 4AP and ensured specific detection of the substrate *p*APP by the enzymatic conversion as will be shown in the following. In a first step, the enzymatic reaction of ALP with *p*APP causing the production of 4AP was investigated with the enzyme in solution. As has been shown above this is possible, as there is response of the photocurrent to the product 4AP, but barely to the substrate *p*APP. As shown in Figure 5 a-c the enzymatic reaction could be detected for all the 3 geometries in which the enzyme was free in solution, as indicated in Figure 2. However, there were significant differences in the response curves. In contrast to the detection of 4AP alone (geometry S_0_) the response in geometry S_2 _for *p*APP in the conversion with ALP is small, probably due to a depletion of the substrate near the electrode because of electrostatic repulsion.

In a final step the enzyme has been immobilized in a single and double layer as depicted in Figure 2d and 2e. By this method, the biospecific recognition element is part of the device and no substances have to be added to the solution despite the molecule to be detected (here *p*APP). In the case of geometry I_2 _the maximum photocurrent response is relatively low (Figure 5e). This corresponds directly to the control experiments in which 4AP has been detected directly (Figure 4e). The ALP/polyelectrolyte layers seem to hinder diffusion of 4AP to the QDs surface. Immobilization of ALP also reduces the steepness of the dose-response curve (*cf*. Figure 5a,b versus Figure 5d). Nevertheless, for electrodes with a single layer of ALP fixed with the polyelectrolyte PAH a very well defined response to the enzyme substrate is obtained. This shows that the concept of a photobioelectrochemical sensor can be realized with the example of ALP. Sensitivity for 4AP detection could be provided in the range from 25 μM to 1.5 mM (*cf*. Figure 3, in all geometries shown, addition of 25 μM clearly triggered a response in the photocurrent). We want to point out that the aim of this paper was not the development for a practical sensor for direct *p*APP detection in real samples, but rather to demonstrate the proof of concept for a photo-triggered enzyme sensor (of the first generation). In order to further analyze the response behavior quantitatively, the dose response curves were fitted with the Michaelis-Menten equation, cfg. Eq. 1 [51]. Hereby we assumed that the rate of the enzymatic reaction v was proportional to the oxidation current I, and thus v/v_max _= ΔI/ΔI_max_, whereby v_max _is the maximum reaction rate and K_M _is the Michaelis-Menten constant, *cf*. Eq. 1. Values are given in Table 1.

(1) $$\Delta \text{I}∕\Delta {\text{I}}_{\max}=\text{c}\left(p\text{APP}\right)∕\left({\text{K}}_{\text{M}}+\text{c}\left(p\text{APP}\right)\right)$$

In literature K_M _values of 0.48 mM [52] and 0.056 mM [53] have been reported, which are in the same order of magnitude as the values detected in our work with the enzyme in solution. For the sensor configuration developed (I_1_) a larger value can be derived from the experiments. It has to be pointed out that in the case of the polyelectrolyte -fixed enzyme the K_M _value has to be considered as apparent K_M _value since here the concentration of half maximum conversion rate is influenced by the immobilization [54]. Comparison of the ΔI_max _values as obtained for direct detection of 4AP (Figure 4) and detection of 4AP after enzymatic degradation of *p*APP to 4AP shows that both oxidation signals (detected at the same geometry and provided abundance of enzyme) are quite similar. This is in good agreement with the detection principle proposed.

In summary the developed sensor as illustrated in Figure 2d by immobilizing the ALP via the polyelectrolyte PAH, provides the proof of principle for a detection system for the enzyme substrate *p*APP. The analytical performance with a detection regime within the concentration range from 0.025 to 1 mM is relatively poor, so that the here presented device has to be seen as a proof of principle demonstrator rather than as an applicable sensor.

## Conclusions

A light controlled bioelectrochemical sensor for *p*APP has been demonstrated. By using QDs as interlayer on gold, 4AP could be oxidized and thus detected via a corresponding photocurrent in case the QDs were illuminated. Enzymes could be functionally immobilized on the sensor surface. This provides the basis for future spatially resolved measurements [40] by selectively illuminating and reading-out only the area of interest of an electrode which is non-structured, but modified with different immobilized enzyme systems. The approach presented here allows for observing enzymatic reactions which yield 4AP as product. We have demonstrated this for the substrate *p*APP and the enzyme ALP. A crucial point for such measurements is to ensure high local enzyme concentration and specificity for the detection of the enzymatic product. By using a polyelectrolyte layer of PAH, the enzyme ALP could be immobilized on the electrode surface, retaining enzymatic activity. However, polyelectrolyte layers can also hinder diffusion of the molecule to be detected 4AP to the QD surface, thus hindering detection. For this reason permeability of the polyelectrolyte layers has been studied here for the respective molecule.

## Competing interests

The authors declare that they have no competing interests.

## Authors' contributions

WK, GG, DH and JMM: performed experiments and analyzed data. PRG: designed experiments and analyzed data. FL and WJP: designed experiments and wrote manuscript. All authors read and approved the final manuscript.

## Supplementary Material

Additional file 1 **Supporting information: Cleaning of gold electrodes**. Immobilization of QDs on the electrode surface. Confirmation of QDs immobilization. Detection of 4-aminophenol and *p*-aminophenyl phosphate (*p*APP). Immobilization of ALP in polyelectrolyte layers on top of the QDs layer. Set-up for the detection of fotocurrents [55-57]. Click here for file
