# Supplementary material for: Light triggered detection of aminophenyl phosphate with a quantum dot based enzyme electrode
Source: J Nanobiotechnology. 2011 Oct 7;9:46. doi: 10.1186/1477-3155-9-46 (PMC3204279; doi:10.1186/1477-3155-9-46)
Supplement: Additional file 1 — Supporting information: Cleaning of gold electrodes. Immobilization of QDs on the electrode surface. Confirmation of QDs immobilization. Detection of 4-aminophenol and p-aminophenyl phosphate (pAPP). Immobilization of ALP in polyelectrolyte layers on top of the QDs layer. Set-up for the detection of fotocurrents [55-57]. [file 1477-3155-9-46-S1.DOC]

Waqas Khalid^1^, Gero Göbel^2^, Dominik Hühn^1^, Jose Maria Montenegro^1^, Pilar Rivera Gil^1^, Fred Lisdat^2^, Wolfgang J. Parak^1*^

^1^Fachbereich Physik and WZMW, Philipps Universität Marburg, Germany

^2^ Biosystems Technology, University of Applied Sciences Wildau, Wildau, Germany

^*^corresponding author: wolfgang.parak@physik.uni-marburg.de

Light triggered detection of aminophenyl phosphate with a quantum dot based enzyme electrode

SUPPORTING INFORMATION

(1) Cleaning of Gold Electrodes

(2) Immobilization of quantum dots (QDs) on the electrode surface

(3) Confirmation of QDs immobilization

(4) Detection of 4-aminophenol (4AP) and *p*-aminophenyl phosphate (*p*APP)

(5) Immobilization of ALP in polyelectrolyte layers on top of the QDs layer

(6) Set-up for the detection of photocurrents

(1) Cleaning of Gold Electrodes.

Gold electrodes were prepared by sputtering first 20 nm film of TiO_2_ followed by a 100 nm film of Au on glass slides. After sonication in toluene for five minutes eventual oxide layers on top of the gold surface were removed by cyclic voltammetry (CV) in 0.1 M NaOH from -0.8 to +0.2 V, following by CV in 0.5 M H_2_SO_4_ from -0.2 to +1.6 V. After CV the gold electrodes were rinsed vigorously with water and then dried with the nitrogen flow. CV traces are shown in Figure SI-1.


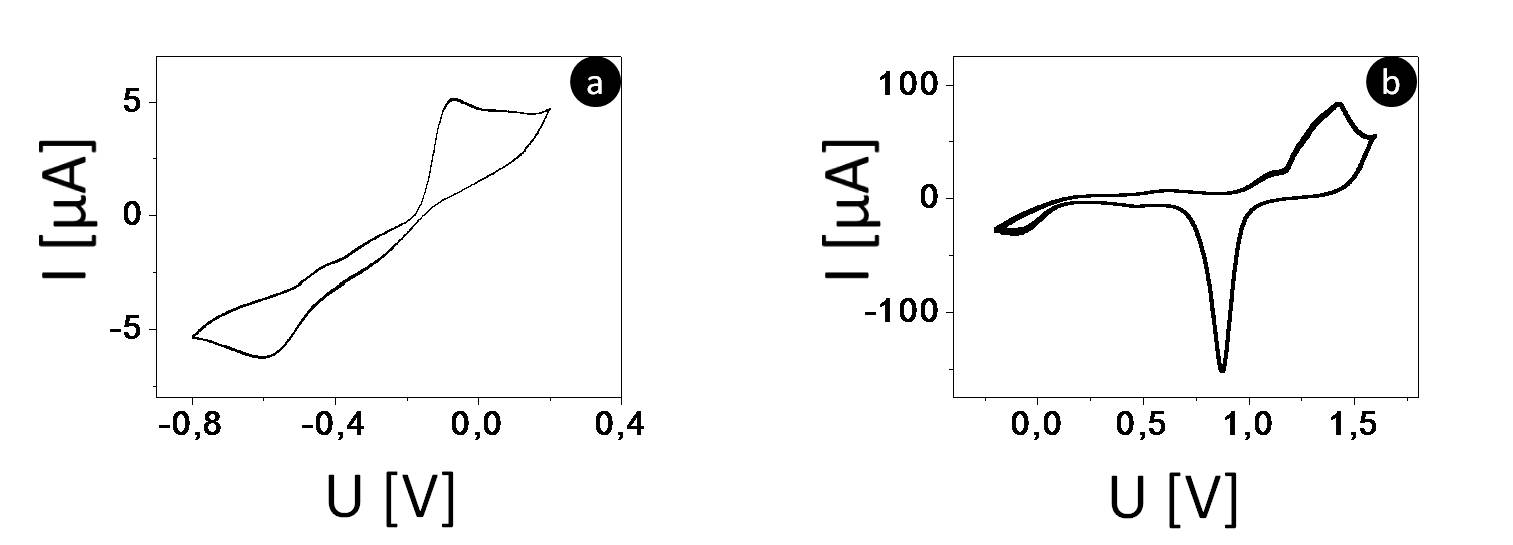


Figure SI-1: Cyclic voltammetry of bare gold electrodes in (a) NaOH and (b) H_2_SO_4_. The current I is plotted versus the applied bias voltage U (versus an Ag/AgCl reference electrode). No illumination / lock-in dectection was used.

(2) Immobilization of quantum dots (QDs) on the electrode surface

In a first step 1,4- benzenedithiol (BDT) molecules were used as the anchoring support for QDs on the gold surface. Self assembled monolayers (SAMs) of BDT were attained by immersing the gold electrodes in 50 mM solution of BDT in toluene for 24 hours[[1]](#footnote-2), see Figure SI-2. BDT contains two SH groups within the benzene ring at position 1 and 4. It is intended that one of the SH groups sticks to the gold surface, while the other one assists the attachment of QDs.


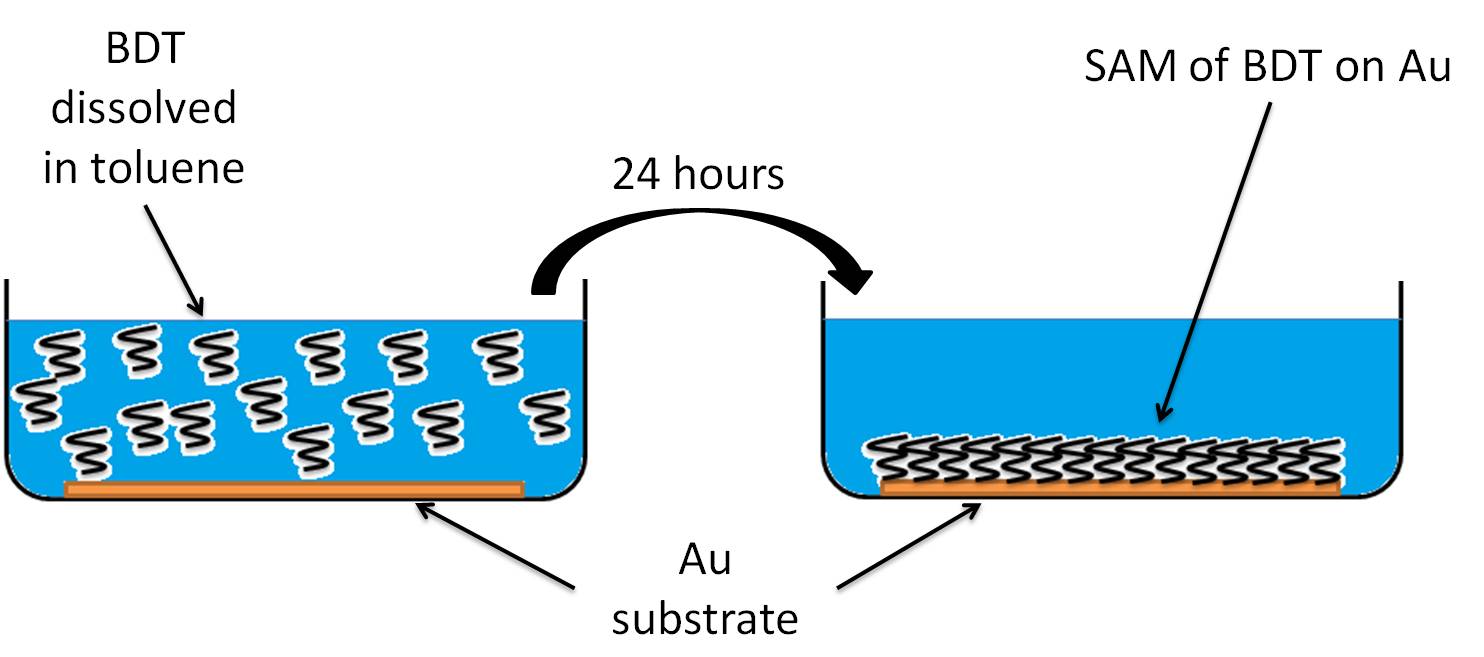


Figure SI-2: Coating of the Au surface with a SAM of BDT.

CdS QDs were synthesized using the protocol established by Kudera et al. [42] using an organic solvent based synthesis, leaving their surface coated with trioctyl phosphine oxide (TOPO). The average diameter of these QDs was around 3 nm with λ_abs_ = 383 nm. During attachment of the QDs to the BDT layer a local ligand exchange of TOPO to the thiols (SH) of the BDT is expected to take place. For attachment of QDs to the surface of BDT coated Au electrodes, 3 methods were explored. For the first method QDs were immobilized via immersion of the BDT immobilized gold electrode in a QDs solution in toluene for 48 hours, see Figure SI-3.

**
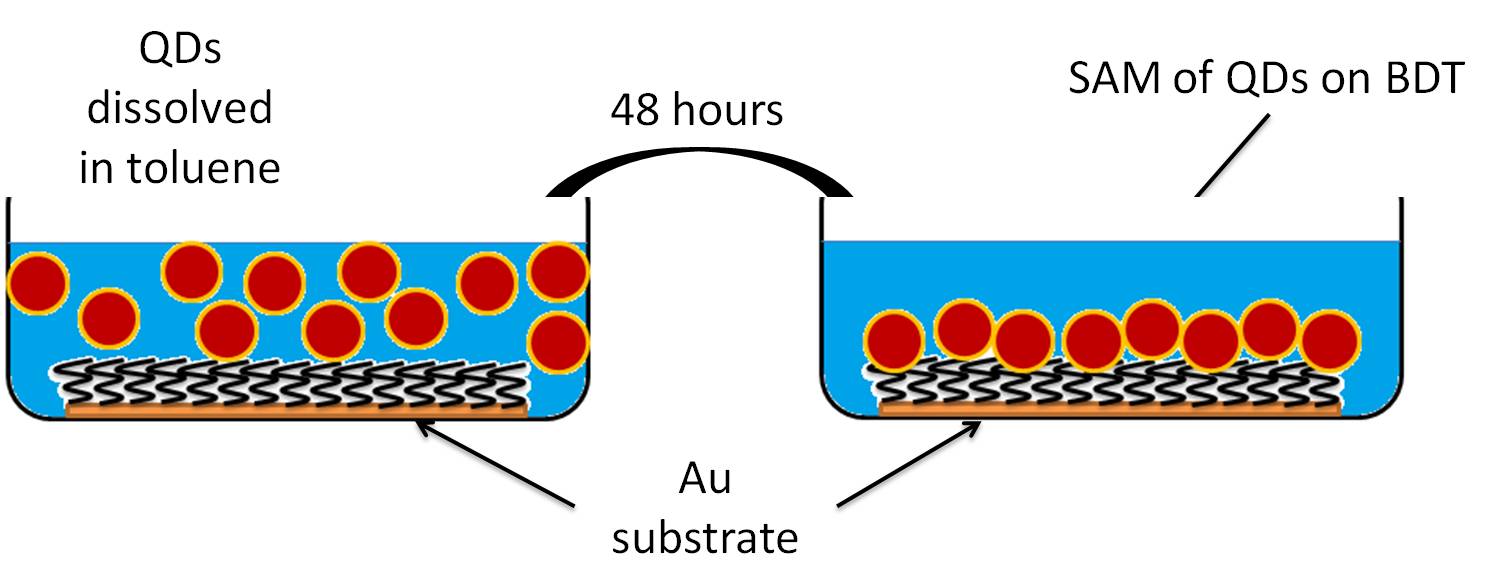
**

Figure SI-3: BDT-coated Au electrodes are immersed for 48 hours in a QDs / toluene solution.

However, during the experiments with QDs layers prepared in this way we observed a significant loss of photocurrent, which we assumed to be due to a loss of QDs from the surface of the homogeneous layers. The second method of immobilization of QDs spin coating was employed, see Figure SI-4. The BDT coated gold electrodes were mounted on the vacuum holder of a spin coated and a spin coated with QDs (dissolved in toluene, c ≈ 136 μM) at 6000 rpm for 3 minutes [43]. For all following measurements this second QDs immobilization method was used. We had also tried to further stabilize the spin coated QDs layer by an additional very thin spin-coated layer of photo-resist on top of the QDs. However, while this layer enhanced the stability of the photocurrent, the sensitive to analytes such as 4-aminophenol (4AP) or hydrogen peroxide (H_2_O_2_) was lost. So this method was not used.

**
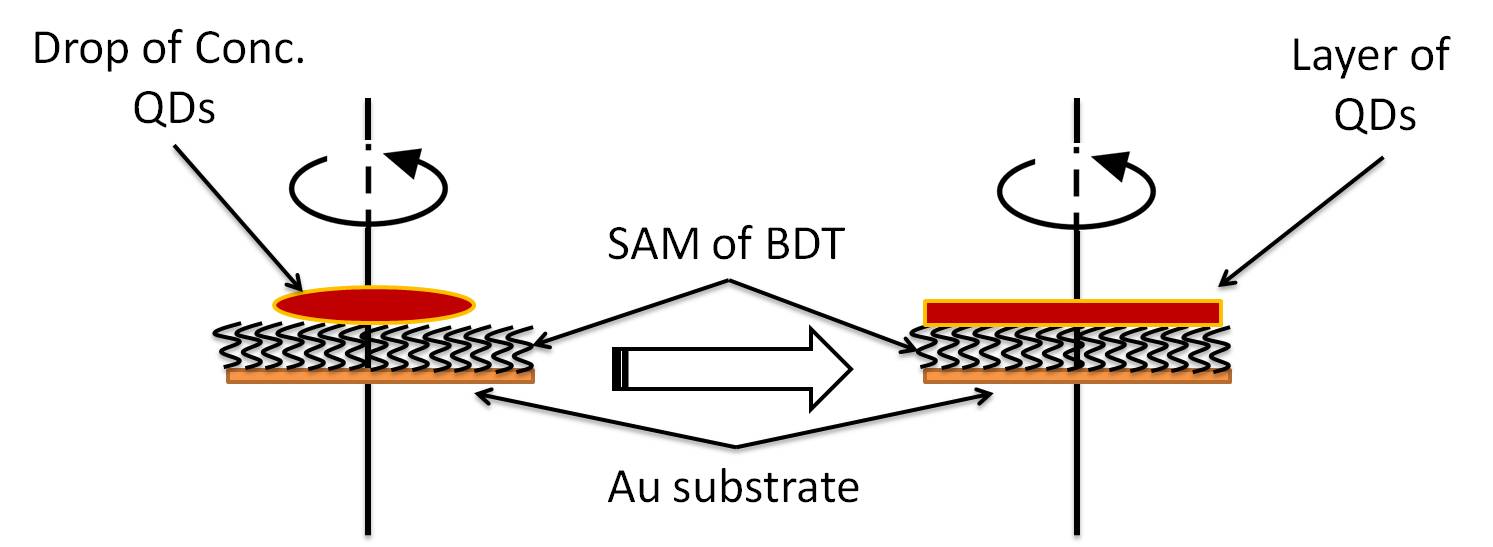
**

Figure SI-4: Spin-coating of QDs on top of BDT-coated Au electrodes.

(3) Confirmation of QDs immobilization

Immobilization of QDs on BDT coated Au electrodes was confirmed with i) CV in hexacyanoferrate solution and ii) via photocurrent detection. In Figure SI-5 voltammograms recorded in 0.1 M phosphate buffer and with ferri / ferrocyanide (50 mM Fe(CN)_6_^3-^ and 50 mM Fe(CN)_6_^4-^) are shown. On the bare Au electrode the characteristic oxidation and reduction peaks are visible (E^o^ = 0.361 V vs. NHE at 25 ºC). At positive bias U around 0.4 V Fe(II) is oxidized to Fe(III). In contrast, virtually no oxidation or reduction peaks were recorded for the Au electrodes with BTD / QDs layer. In addition there was a decrease of the charging current of about 30 times compared to bare Au electrodes, indicating the presence of the BDT / QDs layer.


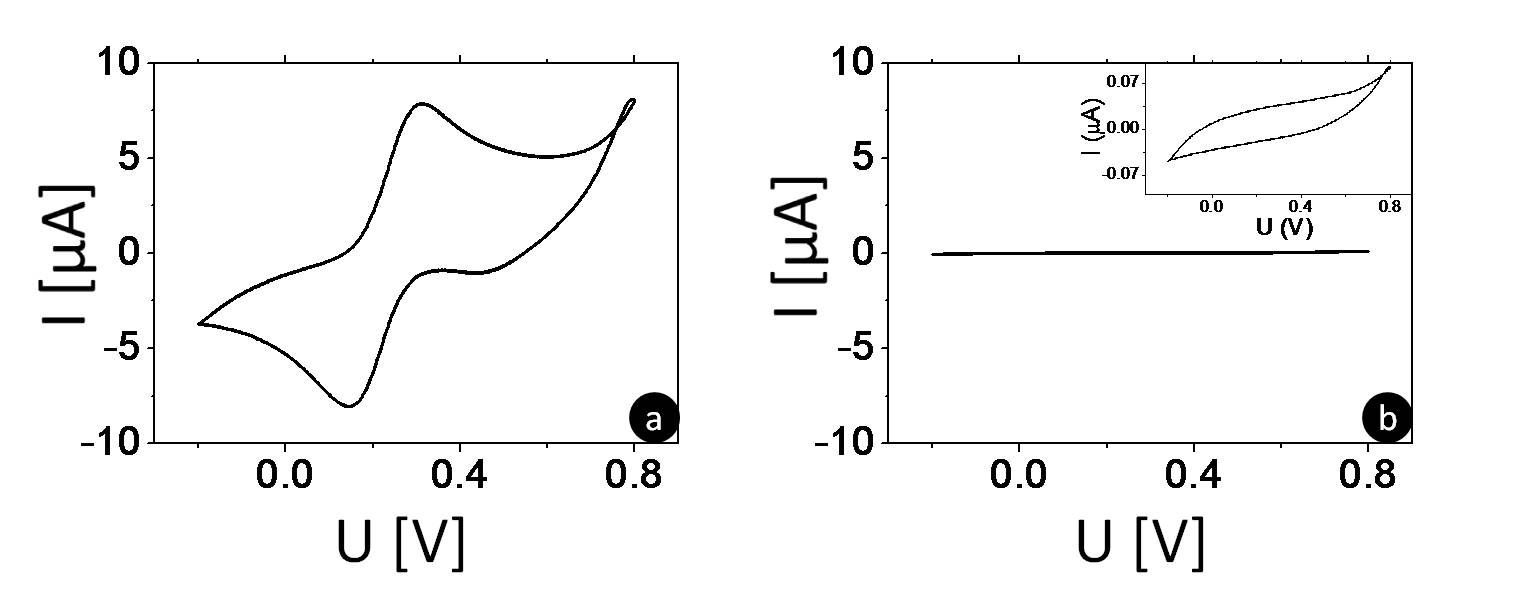


Figure SI-5: Cyclic voltammograms in phosphate buffer and hexacyanoferrate (a) with bare Au electrodes and (b) with QDs immobilized on BDT-coated Au electrodes. No illumination / lock-in dectection was used.

Alternatively we confirmed QD immobilization via photocurrent detection. In Figure SI-6 the currents upon illumination are shown at an applied bias potential of U = +200 mV. In Figure SI-6 (a) pulses of light are illuminated on bare Au electrode but no photocurrent can be observed, however upon illuminating the light pulses on QDs immobilized Au electrodes photocurrent can be observed as shown in Figure SI-6 (b). This goes on to show that a layer of QDs is located on Au surface.


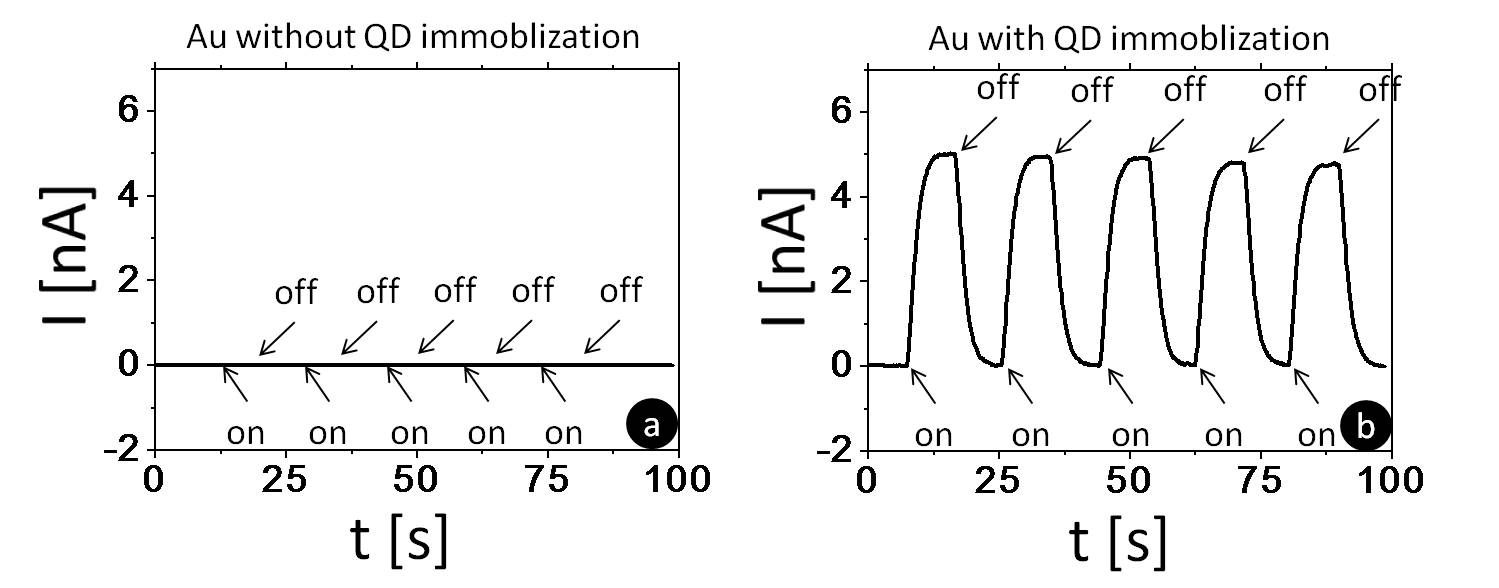


Figure SI-6: Current I recorded at fixed bias U = +200 mV of BTD-coated Au electrodes without (a) and with (b) immobilized QDs. Illumination was switched on and off. Currents were detected with the help of a lock-in amplifier [43] (modulation of light source f = 23.8 Hz, time constant of lock-in τ = 1 s).

(4) Detection of 4-aminophenol (4AP) and *p*-aminophenyl phosphate (*p*APP)

4-aminophenol (4AP) is the product of the enzymatic reaction of the enzyme alkaline phosphatase (ALP) and its substrate *p*-aminophenyl phosphate (*p*APP). 4AP is not very stable [55]. It oxidizes quickly under light and regular atmosphere conditions.

For this reason 4AP was dissolved in 25% methanol and 75% buffer at pH = 5 at room temperature, and also measurements were carried out at room temperature. As oxidation of 4AP was significantly reduced under these conditions all following experiments were carried out with this way of 4AP preparation. Only under these conditions preventing self oxidation of 4AP the 4AP signal was significantly higher than the one for *p*APP[[2]](#footnote-3).

4AP was detected upon oxidation on the surface of illuminated QDs. For this purpose a bias favoring oxidation have to be chosen. Thus the bias at which the maximum oxidation current could be generated under illumination had to be determined. As a result for all following measurements a bias U = +200 mV was chosen, as oxidation of 4AP was found to be maximum at this voltage.


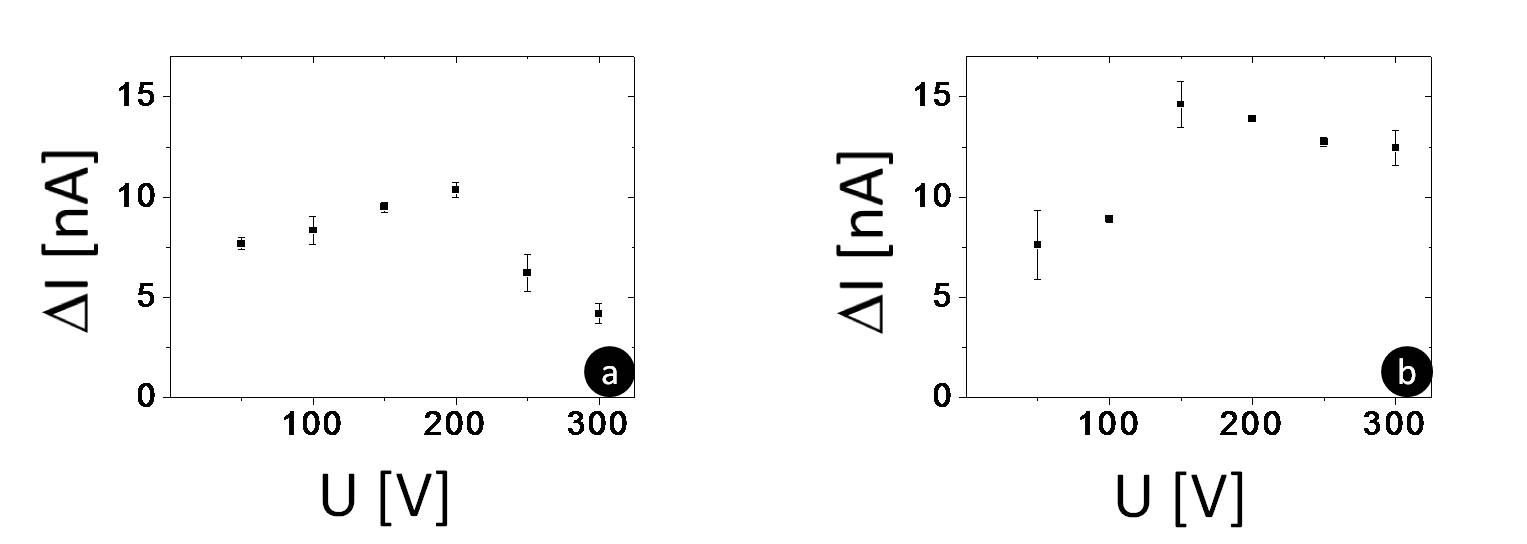


Figure SI-7: Oxidation current ΔI in dependence of the applied bias U (versus an Ag/AgCl reference electrode). As bath solution 1.22 mM 4AP in 0.1 M phosphate buffer (pH = 7.8) was used. Measurements were performed for geometry S_0_ (a) and S_1_ (b). The data points correspond to averages of currents as recorded from two different electrodes. Currents were detected with the help of a lock-in amplifier [43] (modulation of light source f = 23.8 Hz, time constant of lock-in τ = 1 s).

*p*APP was synthesized following the protocol from Frew et al. [56] . For this purpose *p*-nitrophenyl phosphate (*p*NPP) was used, which is another substrate for enzyme ALP [57]. 10 g of *p*NPP was dissolved in 23.81 ml of distilled water and the pH was adjusted to 9 by addition of 10% NaOH. Then 21.71 g of Na_2_S•9H_2_O was added and the solution was heated to 90-95 °C for 1 hour. The solution was then allowed to cool down. After cooling, concentrated HCl was added to obtain a very low pH ~ 0. The solution was allowed to cool down and the pH was then adjusted to 4-5 with 25% NaOH. Then, the solution was filtered and the filtrate was washed with boiling methanol. The product was finally obtained in the form of crystals. *p*APP is a much better substrate for ALP than *p*NPP [34], and it can be detected at much lower potential than *p*NPP, which allows for reducing noise caused by high bias potentials.

Dose response curves for 4AP and *p*APP for different geometries are shown in Figure SI-8 and Figure SI-9. In geometry S_2_ the dose response curve for *p*APP differed from the expected shape. Since the polyelectrolyte layers are charged there might be the possibility of some impurity molecules accumulating at the charged PSS layer. It was noteworthy however that only the polyelectrolyte PSS was causing this, as PAH immobilized in geometry S_1_ did not have any effect. As control for impurities besides *p*APP as synthesized in our group also *p*APP from Diagno Swiss was used, which yielded the same results.


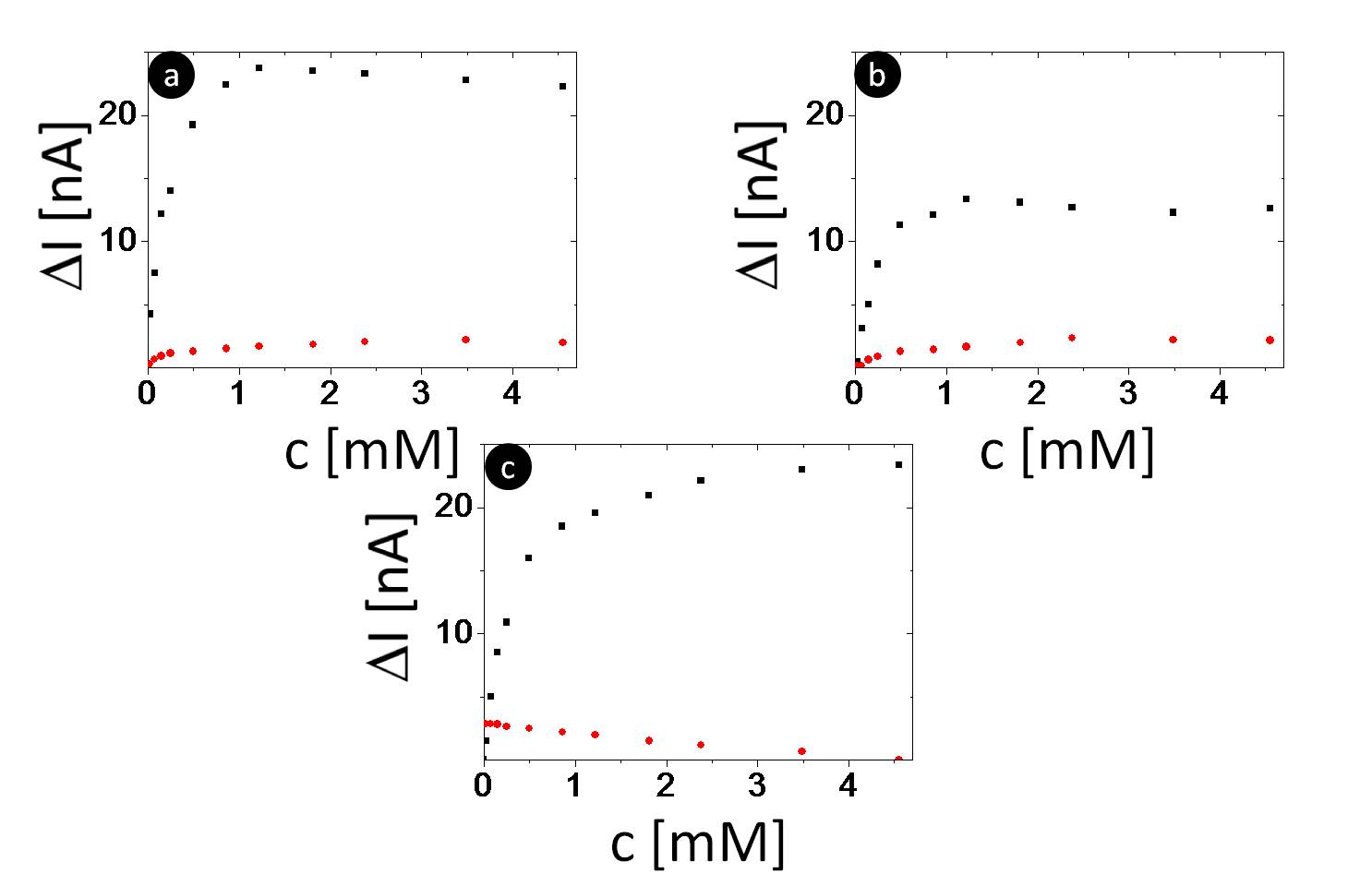


Figure SI-8: Dose response curves for 4AP (black) and *p*APP (red) measured in 0.1 M phosphate buffer with pH 7.8 for geometries S_0_ (a), S_1_ (b), S_2_ (c). Currents were detected with the help of a lock-in amplifier [43] (modulation of light source f = 23.8 Hz, time constant of lock-in τ = 1 s).


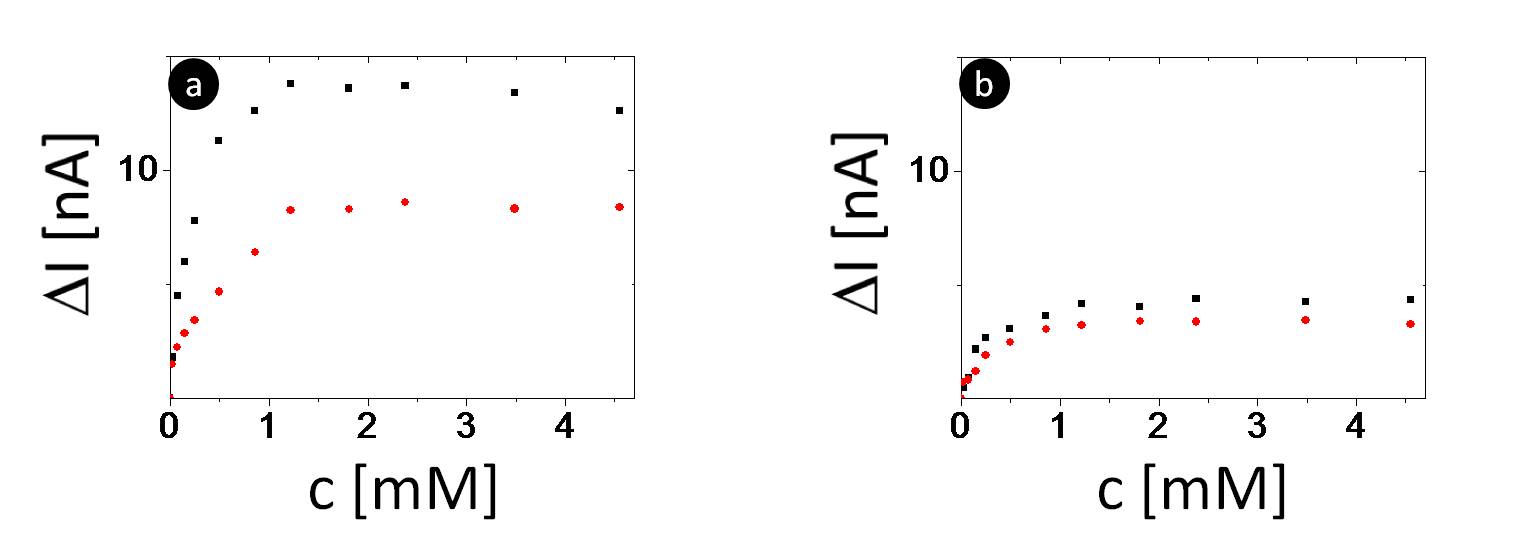


Figure SI-9: Dose response curves for 4AP (black) and *p*APP (red) measured in 0.1 M phosphate buffer with pH 7.8 for geometries I_1_ (a), and I_2_ (b). Currents were detected with the help of a lock-in amplifier [43] (modulation of light source f = 23.8 Hz, time constant of lock-in τ = 1 s).

(5) Immobilization of ALP in polyelectrolyte layers on top of the QD layer

In order to observe attachment of the enzyme ALP to the PAH layers we conjugated ALP with FITC dye. As shown in Figure SI-11 we observed that ALP conjugated with FITC caused an increasing fluorescence signal with increasing number of PAH + ALP double layers (geometries I_i_, with the number of PAH + ALP double layers i).


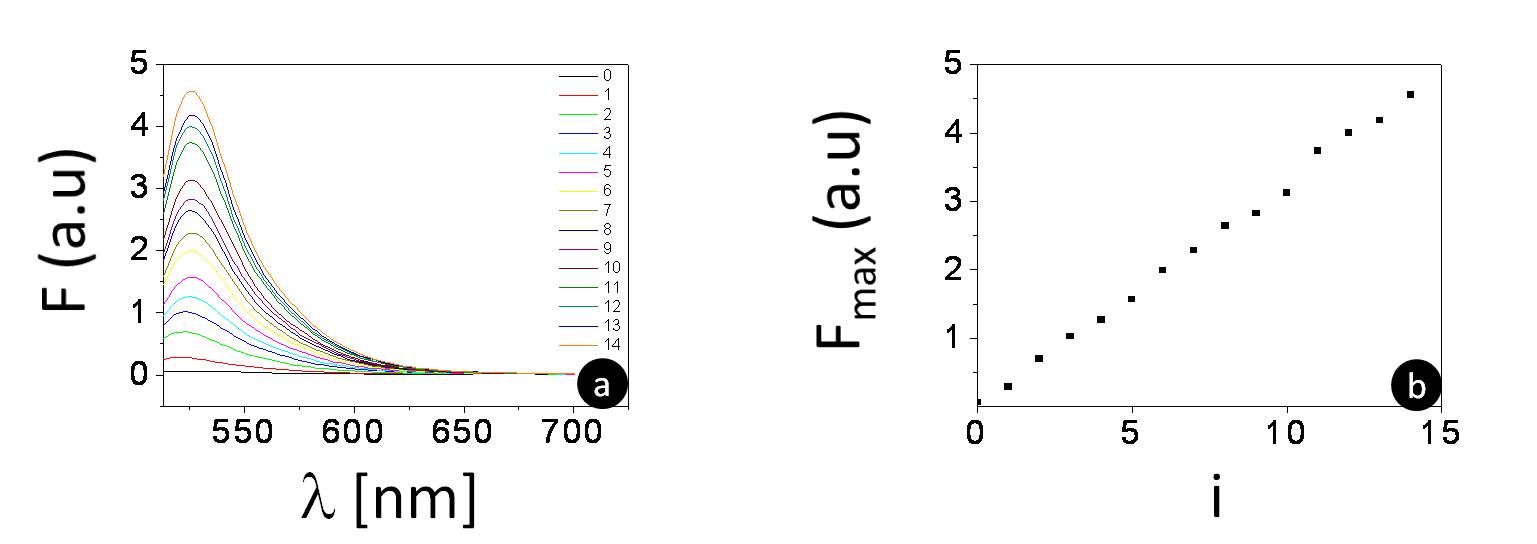


Figure SI-10: a) Fluorescence spectra F(λ) of electrodes with i PAH + ALP double layers. The fluorescence is due to FITC labeling of ALP. b) Maximum of fluorescence plotted versus the number of PAH + ALP double layers i.

(6) Set-up for the detection of photocurrents

Au Electrodes: Gold chips were prepared by sputtering first 20 nm film of TiO_2_ on glass slides followed by a 100 nm film of Au. Chips were then cut to 7 mm × 7 mm chip. For electrical connection a wire was soldered on one edge of the chip, cfg. Figure SI-13.


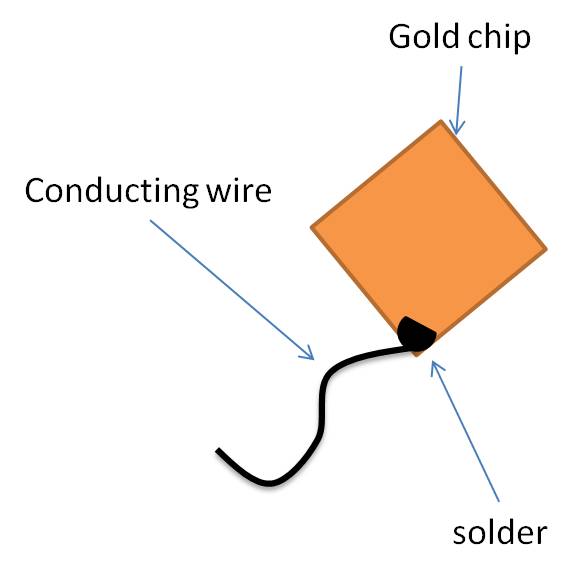


Figure SI-13: Sketch of Au chips

Electrochemical measurement cell: A sketch of our electrochemical measurement cell which is placed on top of the Au chips is shown in Figure SI-14. It comprises a hollow cylinder, which contains the bath solution and builds a support for both, the reference and the counter electrode. The bath solution can be exchanged via an outlet. Light is entering the chamber from the top to hit the gold chip on the bottom. At the bottom the cylinder is closed besides a small hole with a radius of 2.5 mm. The cylinder is tightly sealed on top of the gold chip with an O-ring.


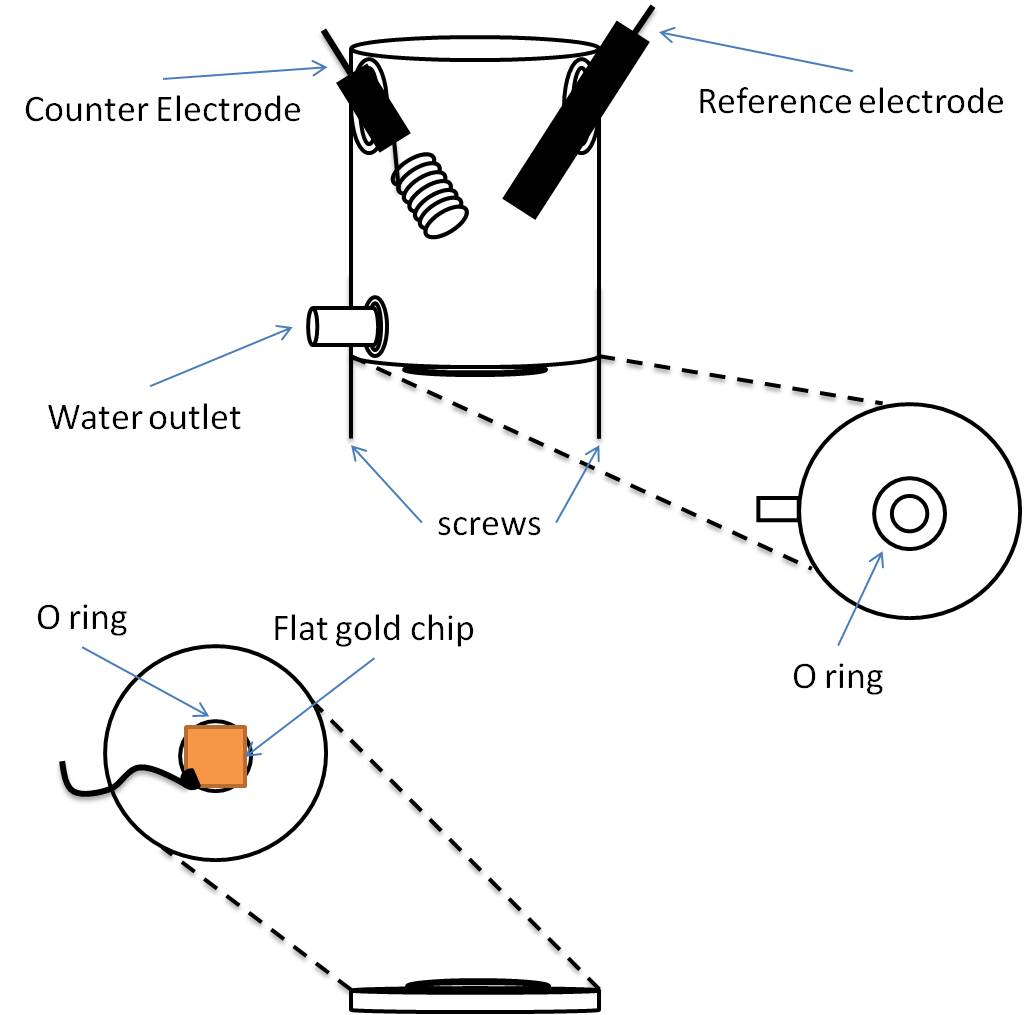


Figure SI-14: Schematics of the home made electrochemical measurement cell.

Light Source: As light source a Xe arc lamp with λ_em_ = 300 – 700 nm, controlled by a lamp power supply LPS 220 by Photon Technology International was used.

Lenses for focusing the light pointer: To focus the light from the arc lamp to the electrochemical cell a convex and a plano-convex lens along with a 45° mirror were used as shown in Figure SI-16. All three components were purchased from Linos Germany.

Optical chopper: An optical chopper (Scitec instruments) was introduced in the light path before the lens to modulate the incident light at a desired frequency.

Lock-in Amplifier: An EG&G lock-in amplifier (Model # 5210) was used to improve the signal to the noise ratio. The reference frequency for the lock in was the modulation frequency used for the chopper. The lock-in was connected to the current output I of the three electrode system. The output of the lock in was interfaced with a PC and read by a serial port reader, cfg. Figure SI-16.

Three electrode system: In the three electrode system the operational amplifier provides a constant potential at which working electrode (WE, in our case the Au chip) is set to ground. A fixed potential is applied to the reference electrode (RE, in our case the Ag/AgCl electrode). The counter electrode (CE, a platinum wire) lies at a higher or lower potential in comparison to the WE. The CE generates the current by oxidizing or reducing the redox pairs in solution.


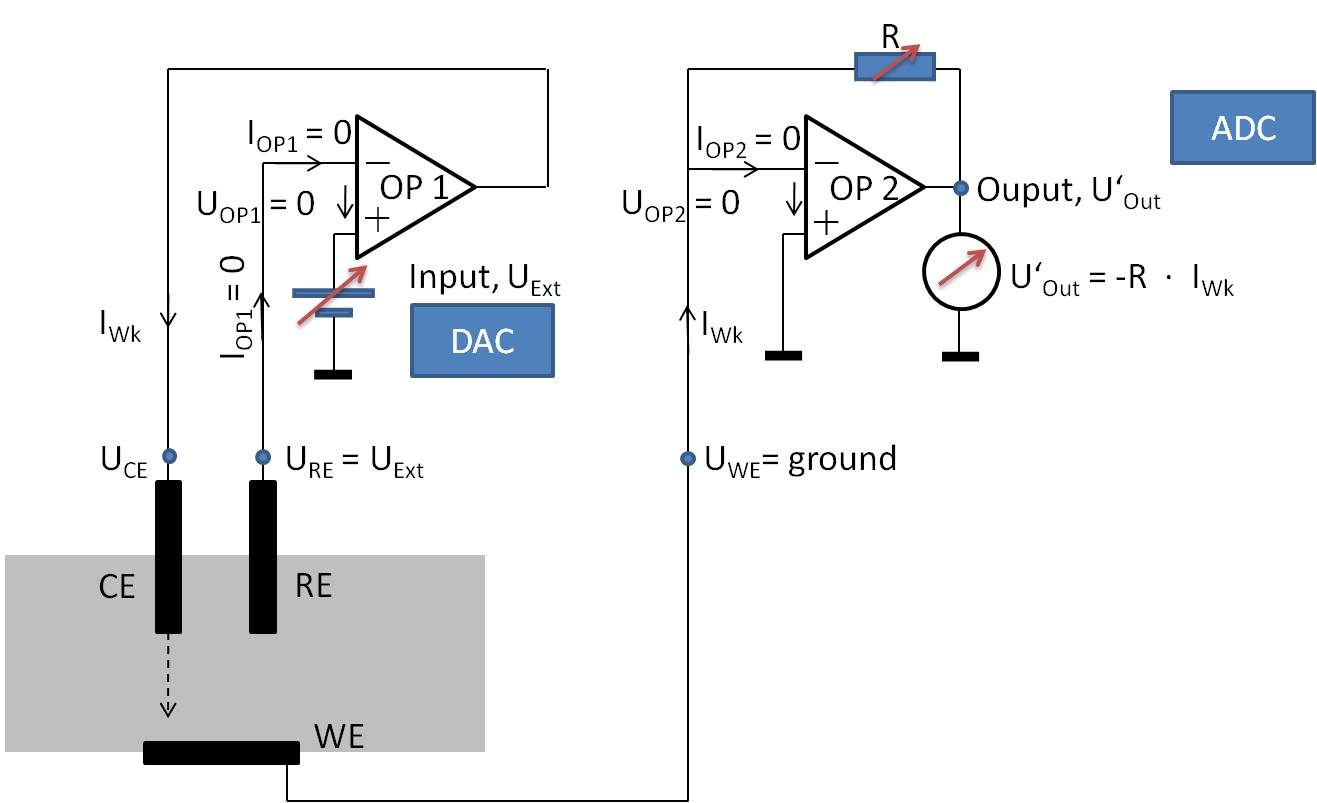


Figure SI-15: Schematics of a three electrode system

The essential components of our system were three electrodes and a microcomputer with an interface card for digital to analog (DAC) and analog to digital (ADC) conversion. A Ag/AgCl saturated RE, a spiral like platinum CE, and a gold chip WE were assembled within the electrochemical cell as shown in Figure SI-14. Two operational amplifiers OP1 and OP2 were mounted within the three electrode system. The voltage U_Ext_ (= the bias voltage U in the cyclic voltammograms) was applied by the DAC at the + input of OP1. The RE was directly connected with the – input of OP1. No current flows through the RE and I_OP1_ is zero. The purpose is to ensure that the voltage difference between the RE and WE always remains constant (at bias voltage U = U_ext_). OP2 has a variable resistor mounted between the - input and output of OP2 to increase the sensitivity of the instrument. This arrangement is called current to the voltage converter. The output of OP2 is measured with the ADC or the lock-in amplifier. The output is proportional to the current I = I_Wk_ through the WE and CE. A ± 2 V input and output range is set for the DAC and ADC lab board. Input and output resolution was 16 bit.

Block Diagram: The electrochemical cell and the three electrode system were placed into a Faraday’s cage to shield against external static electric fields. The light from the light source was focused with a convex lens. In between the convex lens and the light source a chopper was installed to modulate the light at desired frequency. The incident light was aligned after passing through the lens. The resulting light beam was focused with the help of a plano-convex lens on the surface of the QD coated Au chip inside the electrochemical measurement cell


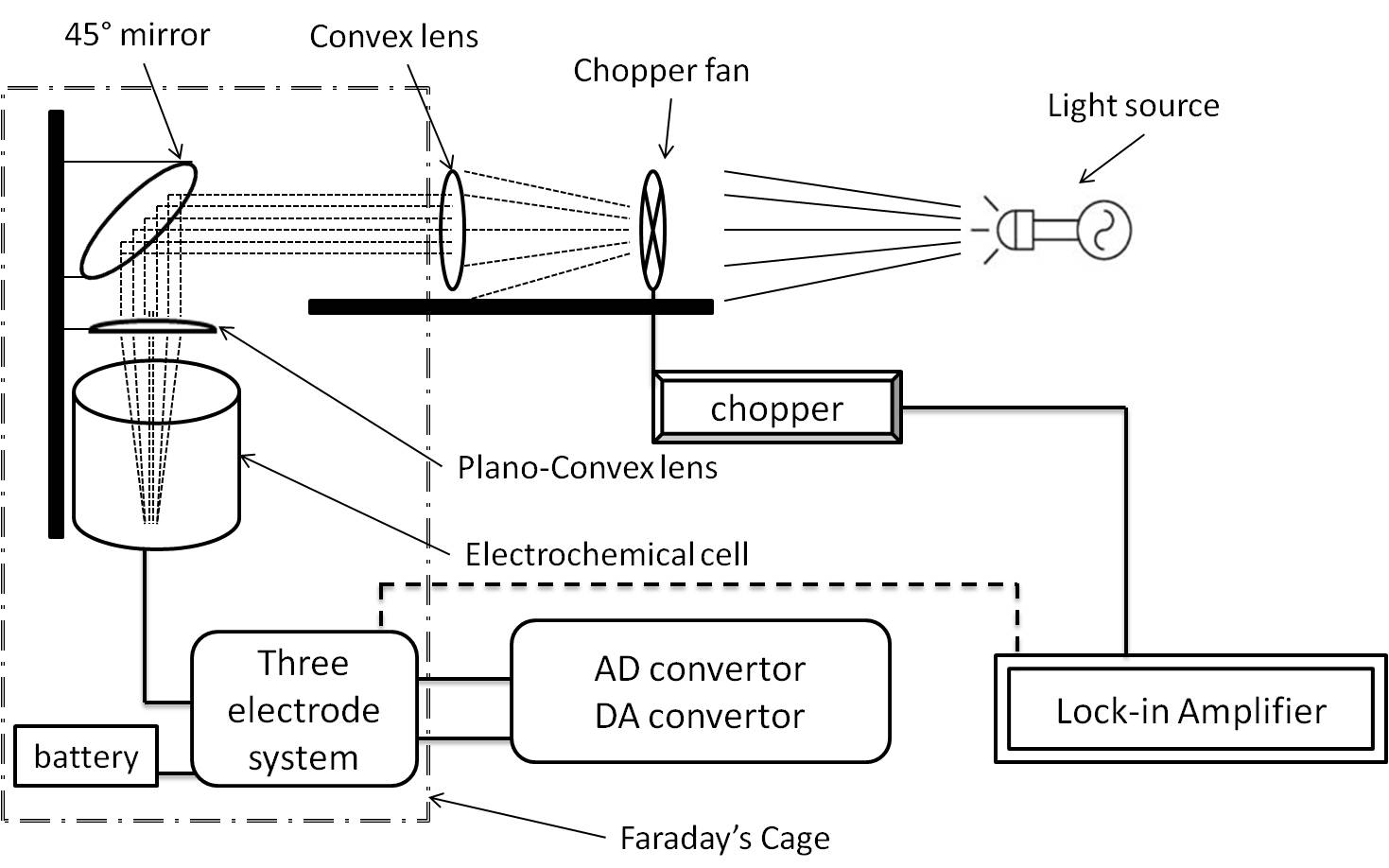


Figure SI-16: Block diagram of the setup

1. We also tried to immobilize BDT via spin coating, which however did not yield good results. [↑](#footnote-ref-2)
2. [↑](#footnote-ref-3)
